# Calculation of Average Mutual Information (AMI) and False-Nearest Neighbors (FNN) for the Estimation of Embedding Parameters of Multidimensional Time Series in Matlab

**DOI:** 10.3389/fpsyg.2018.01679

**Published:** 2018-09-10

**Authors:** Sebastian Wallot, Dan Mønster

**Affiliations:** ^1^Max Planck Institute for Empirical Aesthetics, Frankfurt, Germany; ^2^Interacting Minds Centre, School of Culture and Society, Aarhus University, Aarhus, Denmark; ^3^Department of Economics and Business Economics, Aarhus University, Aarhus, Denmark; ^4^Department of Management, Aarhus University, Aarhus, Denmark

**Keywords:** average mutual information, false-nearest neighbors, time-delayed embedding, Multidimensional Time series, Multidimensional Recurrence Quantification Analysis, code:Matlab

## Abstract

Using the method or time-delayed embedding, a signal can be embedded into higher-dimensional space in order to study its dynamics. This requires knowledge of two parameters: The delay parameter τ, and the embedding dimension parameter *D*. Two standard methods to estimate these parameters in one-dimensional time series involve the inspection of the Average Mutual Information (AMI) function and the False Nearest Neighbor (FNN) function. In some contexts, however, such as phase-space reconstruction for Multidimensional Recurrence Quantification Analysis (MdRQA), the empirical time series that need to be embedded already possess a dimensionality higher than one. In the current article, we present extensions of the AMI and FNN functions for higher dimensional time series and their application to data from the Lorenz system coded in Matlab.

## 1. Introduction

Many prominent methods of nonlinear time series analysis, such as Recurrence Quantification Analysis (RQA – Webber and Zbilut, 1994) or Convergent Cross Mapping (Sugihara et al., 2012; Mønster et al., 2017) require the reconstruction of the phase space profiles of time series, because the analysis techniques are applied to the phase-space profiles of the time series, and not to the time series themselves.

One way to solve the problem of reconstructing a higher dimensional phase-space from a one dimensional time series is the method of time-delayed embedding (Packard et al., 1980; Takens, 1981): If the dynamics of the latent dimensions that co-determine the dynamics of an observed time series are coupled to each other, then one can reconstruct the dynamics of these latent dimensions from the observed one-dimensional time series by plotting the values of that series (multiple times) against itself at a certain lag, as shown by Takens' theorem. The resulting coordinates in higher dimensional phase-space approximate the phase-space of the actual multidimensional system from which the original time series was taken.

**Figure 1:**
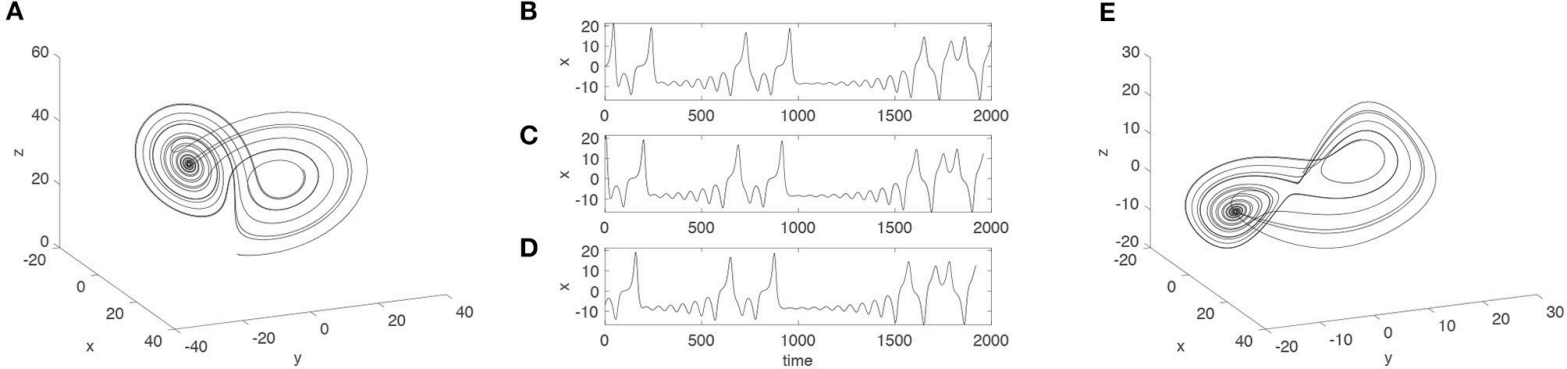
The original three-dimensional Lorenz system **(A)**, a time series corresponding to the dynamics of the *x*-axis of the Lorenz system **(B)**, two surrogate series of the *x*-axis data by shifting the time series for a number of lags equal to τ **(C)** and 2τ **(D)**. Note the loss of data points in creating the surrogate data in **(C,D)** evident through missing data points at the end of the time series. When the time series in **(B–D)** are plotted against each other, the resulting phase-space **(E)** approximates the original phase-space of the Lorenz system **(A)**.

In order to perform phase-space reconstruction using the method of time-delayed embedding, one needs to know two parameters: The delay parameter τ, which is the lag at which the time series has to be plotted against itself, and the embedding dimension parameter *D*, where *D* − 1 is the number of times that the time series has to be plotted against itself using the delay τ. If these two parameters are known, one can reconstruct an approximation of the original phase-space dynamics from a one-dimensional time series (Buzug and Pfister, 1992). Figure 1 provides an example using data from the Lorenz system (Lorenz, 1963), which is a system of three coupled differential equations. For the Lorenz system, *D* is principally known (i.e., *D* = 3, except for the fix-point attractor of the system), but τ still needs to be estimated, because it depends on the properties of numerical integration method chosen.

For most empirical time series, however, both of these two parameters are unknown ab initio and have to be estimated. Two standard methods to estimate these parameters in one-dimensional time series are the computation of the Average Mutual Information (AMI) function and the False Nearest Neighbor (FNN) function, where the first local minima of those functions (or the point at which those functions level-off) are indicative of the delay and embedding dimension (e.g., Abarbanel, 1996; Marwan et al., 2007).

Accordingly, these functions have been implemented in many software packages for the analysis of dynamic systems. Sometimes, however, more than one time series is available (i.e., different variables from the same system, or the same variables from different parts of the system), and the basis for the analysis—and the reconstruction of the phase-space—is multidimensional time series data. For example, we recently described multivariate extensions of RQA, namely Multidimensional Recurrence Quantification Analysis (MdRQA: Wallot et al., 2016b) for the analysis of multidimensional time series and Multidimensional Cross-Recurrence Quantification Analysis (MdCRQA: Wallot, 2018). Hence, when the underlying data is multivariate, then one needs to somehow combine the information from the individual component signals of the multidimensional time series to properly estimate values for τ and *D*.

In psychology, the interest in examining the common dynamics of multidimensional/multivariate time series has been particularly prominent in psycho-physiological research, particularly on the physiological signature on arousal and emotions (see (Kreibig, 2010), for a review) and in joint action research (see (Marsh et al., 2009; Knoblich et al., 2011), for reviews)—or the intersection of both sub-fields (e.g., Konvalinka et al., 2011; Müller and Lindenberger, 2011; Mønster et al., 2016).

Analysis methods that are based on phase-space reconstruction have been particularly prominently used in research on joint action (e.g., Shockley et al., 2003, 2007; Richardson and Dale, 2005; Dale et al., 2011; Fusaroli and Tylén, 2016; Mønster et al., 2016; Wallot et al., 2016a), but their application was thus far constrained to the assessment of two one-dimensional time series. For example, Louwerse et al. (2012) investigated multimodal facial expression during conversation, such as smiling, frowning, nodding, rolling the eyes etc. However, the application of Cross-Recurrence Quantification Analysis (CRQA), a phase-space based analysis technique, could only be used to investigate the shared dynamics for each of those features in isolation between two interlocutors, because CRQA is restricted to one-dimensional time series. As noted above, we have recently published multivariate extensions that would allow the simultaneous analysis of multiple features, but proper methods for the parameter estimation of the analysis were missing.

The assessment of coupling or common dynamics between multidimensional time series is relevant to many fields, but it has mostly been physics and related disciplines that have developed models and parameter estimation procedures for such data. Usually, however, these methods are published as formal descriptions. Accordingly, applicable software—for example for psychologists interested in using such methods—needs to be custom-coded.

Hence, in the present paper, we present Matlab implementations of the AMI and FNN functions used for one-dimensional time series that extend them for the application to multidimensional time series in order to estimate τ and *D*. For the delay parameter τ, we implemented the uniform multivariate average mutual information method (Vlachos and Kugiumtzis, 2009), and for the embedding dimension parameter *D*, we present an implementation that is a simple extension of the original false nearest neighbor algorithm proposed by Kennel et al. (1992).

In the following, we will briefly describe the two methods, then provide an example application using the Lorenz system, summarize the Matlab functions that implement the methods, and finish with a discussion of their advantages and limitations, as well as suggestions for their usage. A summary of the abbreviations and notation used in this article, can be found in the Appendix to this paper.

## 2. Average mutual information (AMI)

To find the optimal time delay for embedding a one-dimensional time series, Fraser and Swinney (1986) developed a method to find time delayed coordinates that are as independent from each other as possible. They quantified dependence as the mutual information *I*(*x*(*t*), *x*(*t* + τ)) between the original time series *x*(*t*) and the time series *x*(*t* + τ) shifted by τ. Since mutual information is computed for a times series and a time-shifted version of the same time series, this is called the auto mutual information or average mutual information (AMI). The auto mutual information can be considered a nonlinear generalization of the autocorrelation function, and it is given by the expression

(1) $$I(x(t),x(t+\tau ))=\sum_{i,j}p_{ij}(\tau )\text{log}\left(\frac{p_{ij}(\tau )}{p_{i}p_{j}}\right)$$

Here, *p*_*i*_ is the probability that *x*(*t*) is in bin *i* of the histogram constructed from the data points in *x*, and *p*_*ij*_(τ) is the probability that *x*(*t*) is in bin *i* and *x*(*t* + τ) is in bin *j*. Note that only the joint probability *p*_*ij*_(τ) depends on τ, and that the AMI function also depends on how the histograms are constructed, i.e., the width and position of the bins.

To obtain coordinates for time delayed phase-space embedding that are as independent as possible, Fraser and Swinney proposed using the position of the first minimum of *I*(*x*(*t*), *x*(*t* + τ)) as the optimal value of τ. Using that particular value means that the first coordinate of the phase-space embedded signal *y*_1_(*t*) = *x*(*t*) will be maximally independent of the second coordinate *y*_2_(*t*) = *x*(*t* + τ), etc. In practice the AMI function may not have a local minimum, but may, e.g., be a monotonically decreasing function of τ. Consequently other criteria have been developed, such as the lowest value of τ for which the AMI function drops below the value 1/*e* (see Kantz and Schreiber, 2004, chapter 9 for a discussion).

Several methods have been proposed to generalize this procedure for estimating the time delay to the case of multidimensional time series (Garcia and Almeida, 2005; Hirata et al., 2006; Vlachos and Kugiumtzis, 2009). Here, we have chosen the simplest possible method, referred to by Vlachos and Kugiumtzis (2009) as the uniform multivariate average mutual information method. Using this method, the time delay τ is estimated using AMI averaged over all the dimensions in the data. The analyses presented by Vlachos and Kugiumtzis (2009), showed that the uniform multivariate method achieves state space reconstruction at a quality comparable to more complicated non-uniform multivariate methods.

Of course this simple approach might not be applicable to all multidimensional data, e.g., if some dimensions have a very different auto mutual information function, this might give a different optimal value for the time delay. The implementation presented here allows a simple check for whether this is the case, viz. the option to plot the AMI for each dimension of the data. If the individual dimensions have very different AMI functions, a more advanced approach might be needed, such as embedding individual dimensions using different time delays, or re-sampling some of the dimensions at a lower rate, unless of course this results in loss of information. Another way might be do a search of the parameter space, where for example the minimum, maximum and average values of the parameters are used, and the results are compared to each other.

Otherwise, the time delay for phase-space embedding of the data can be performed just as in the uni-dimensional case, by identifying the value of τ for which the AMI function drops below 1/*e* (i.e., the fist value of the autocorrelation function that lies below the standard error of the function) or attains its first local minimum.

## 3. False nearest neighbors (FNN)

Suppose we have a one-dimensional time series (such as the data displayed in Figure 1B), and we know—or assume—that these data come from a system with higher-dimensional dynamics (such as the Lorenz system displayed in Figure 1A). Then according to Takens' theorem (Takens, 1981), we can try to reconstruct the higher dimensional dynamics by embedding the original one-dimensional time series, using time-delayed surrogate copies of it. To be precise, we can construct a time series *y*(*t*) of *D*-dimensional points from the original one-dimensional time series *x*(*t*) as follows:

(2) y(t)=(x(t),x(t+τ),…,x(t+(D−1)τ)

Here, both *t* and τ are integers used to index the sampled data, but they can be expressed in units of real time when multiplied by the sampling interval. The time delay τ can be estimated using the AMI-approach described above. The embedding dimension *D* can now be estimated by examining the change in distance between neighboring points in phase-space, as we progressively embed the original time series into higher dimensions.

The basic idea underlying the estimation of embedding dimension using FNN was proposed by Kennel et al. (1992) according to the following logic: Suppose two data points in the one-dimensional time series are close together (e.g., adjacent)—then they are neighbors. Their difference in magnitude provides us with the distance of those neighbors. If we embed the time series once (i.e., into two dimensions) using some time delay τ, then we can use the coordinates of those data points to examine whether the distance between them has changed appreciably. If embedding changes the distance between the neighbors appreciably, then they are dubbed false neighbors, and this indicates that the data need to be embedded further. If their distance does not change appreciably, then they are dubbed true neighbors and embedding leaves the shape of the attractor unchanged, meaning that the current embedding dimension is sufficient. This can be done for consecutively increasing embedding dimensions *D*, and we choose a value for *D* at the point where the number of FNN drops to 0, or subsequent embeddings do not change the number of FNNs or the point before which the number of FNNs starts to increase again.

In practice, not all neighbors are investigated, but as the name implies, the method focuses only on the nearest neighbors, and the definition of what an appreciable change in distance is depends on a distance criterion that needs to be defined by the user of the method (see function descriptions and applications below). Using the formula from Kennel et al. (1992), if we have a *D*-dimensional phase-space and denote the *r*th nearest neighbor of a coordinate vector **y**(*t*) by **y**^(*r*)^(*t*), then the square of the Euclidean distance between **y**(*t*) and the *r*th nearest neighbor is:

(3) $$R_{D}^{2}(t,r)=\sum_{k=0}^{D-1}{\left[x(t+k\tau )-x^{\left(r\right)}(t+k\tau )\right]}^{2}$$

Now applying the logic outlined above, we can go from a *D*-dimensional phase-space to (*D* + 1)-dimensional phase-space by time-delayed embedding, adding a new coordinate to **y**(*t*), and ask what is the squared distance between **y**(*t*) and the same *r*th nearest neighbor:

(4) $$R_{D+1}^{2}(t,r)=R_{D}^{2}(t,r)+{\left[x(t+D\tau )-x^{\left(r\right)}(t+D\tau )\right]}^{2}$$

As explained above, if the one-dimensional time series is already properly embedded in *D* dimensions, then the distance *R* between **y**(*t*) and the *r*th nearest neighbor should not appreciably change by some distance criterion *R*_tol_ (i.e., *R* < *R*_tol_). Moreover, the distance of the nearest neighbor when embedded into the next higher dimension—relative to the size of the attractor—should be less than some criterion *A*_tol_ (i.e., *R*_D + 1_ < *A*_tol_). Doing this for the nearest neighbor of each coordinate will result in many false nearest neighbors when embedding is insufficient, or in few (or no) false nearest neighbors when embedding *is* sufficient.

Now the implementation of the FNN-algorithm presented in this paper simply extends the one-dimensional case by beginning this computation with a multidimensional time series, which is effectively treated as a *d*-dimensional phase-space, were *d* is the number of component variables of the multidimensional time series *x*_1_(*t*), *x*_2_(*t*), …, *x*_*d*_(*t*). Accordingly, embedding does not proceed by increasing the embedding dimension *D* by 1 per step, as in the one-dimensional case, but by *d*, because time-delayed surrogates are themselves already *d*-dimensional. Hence, *D* + 1 in Equation (4) is replaced by (*D* + 1) · *d* in Equation (5).

(5) $$\begin{array}{l} \;R_{D\cdot d}^{2}(t,r)=\sum_{j=1}^{d}\sum_{k=0}^{D-1}{\left[x_{j}(t+k\tau )-x_{j}^{\left(r\right)}(t+k\tau )\right]}^{2}\\ R_{(D+1)\cdot d}^{2}(t,r)=R_{D\cdot d}^{2}(t,r)+\sum_{j=1}^{d}{\left[x_{j}(t+D\tau )-x_{j}^{\left(r\right)}(t+D\tau )\right]}^{2} \end{array}$$

This allows us to estimate the embedding parameter *D* for multidimensional time series, but the logic of selecting a value for *D* remains the same as with one-dimensional time series. Note, however, that in our implementation *D* does not denote the embedding dimension *per se*, but denotes the number of times that the *d*-dimensional time series needs to be embedded. Hence, the embedding a *d* = 3-dimensional time series *D* = 2 times results in a phase-space with *d* × *D* = 6 dimensions.

With the described methods it is possible for researchers to estimate parameters for the embedding of multidimensional time series. This is useful in cases where measurements of multiple variables from the same dynamical system are available. In experimental psychology this could be, e.g., multiple variables from a participant in a in psychophysical or psychophysiological study, or the same variable measured for multiple participants that interact in a joint action study. Using the methods and functions provided here, these variables can be used to embed the systems' dynamics in a higher-dimensional phase space, which is a prerequisite for applying phase-space based methods such as MdRQA (Wallot et al., 2016b) or MdCRQA (Wallot, 2018). Without the ability to use multiple variables for the embedding, researchers would be limited to analyzing one variable at a time. As a case in point, it was demonstrated by Wallot et al. (2016b) that MdRQA can be used to systematically analyze dynamics at different levels—from individual dynamics over dyadic dynamics up to groups of three (and in principle the method can be applied to groups of arbitrary size), and that higher-dimensional dynamics seem to capture group interaction better than the average of the individual or dyadic dynamics.

## 4. Example application: the lorenz system

In the following, we use the mdDelay and mdFnn functions on data from the Lorenz system (Lorenz, 1963), which is defined as:

(6) $$\begin{array}{l} \dot{x}=\sigma (y-x)\\ \dot{y}=(\rho -z)-y\\ \dot{z}=xy-\beta z \end{array}$$

To create the example data, we used the parameters σ = 10, ρ = 28, and β = 8/3. Next, we used the functions on each of the individual dimensions of the Lorenz system (*x*, *y*, *z*), corresponding to standard embedding of one-dimensional time series. Then, we used the functions on each of the three possible pairing of the three dimensions (*xy*, *xz*, *yz*), corresponding to two-dimensional time series for which embedding parameters are sought.

Finally, we also subjected all of the three dimensions (*x, y, z*) as a three-dimensional time series to the functions to estimate embedding parameters.

**Figure 2:**
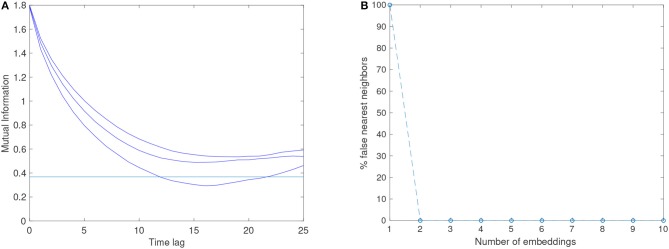
**(A)** Shows the graphical output of the mdDelay function for the three-dimensional time series from the Lorenz system. Since the function was called with the option to show the AMI function (Equation **1**) for each dimension in the data, there are three curves. The default threshold value (1/*e*) is shown as the horizontal line in the plot. **(B)** Shows the graphical output of the mdFnn function for the three-dimensional time series from the Lorenz system. The function was called with the parameters maxEmb = 10, tau = 15 using all three variables *x*, *y* and *z* with 10^4^ number of data points each. The function shows an immediate drop-off of the percentage of false-nearest neighbors to 0, indicating that no additional embedding is necessary for the three-dimensional time series from the Lorenz system.

For example, if we want to use the mdDelay and the mdFnn functions to estimate embedding parameters for the three-dimensional (*x, y, z*) time series consisting of all three dimensions of the Lorenz system, which are stored as columns in the variable data, we use the function mdDelay to estimate the time delay as follows:


tau = mdDelay(data,  'maxLag',  25,
       'plottype',  'all');


Here the maximum time delay has been set to 25 using the optional parameter *maxLag* because the default value of 10 is not big enough, which is evident from the output shown in Figure 2A, where it is clearly seen that the mutual information has not attained a minimum or is below the threshold for delays less than or equal to 10. The parameter *plottype* has been set to “*all,”* meaning that the AMI for each dimension of the data will be shown in the plot.

The first local minimum of the AMI function is for values of τ = 15, 16, and 19 respectively for the three curves. Only for one of the curves does the auto mutual information drop below the default threshold (1/*e*) at τ = 12. The *mdDelay* function uses the threshold criterion per default, but when this fails (as it does for two of the dimensions in the data) it reverts to selecting the minimum. The function calculates the mean value of τ estimated for each dimension, and returns this value, which for the Lorenz data gives a time delay of τ = (12+15+19)/3 ≈ 15.33. Since this value is in good agreement with what is obtained from looking at the plot, we use the nearest integer value τ = 15 to estimate the embedding dimension using the *mdFnn* function, which is called as follows:


[fnnPerc, embTimes] = mdFnn(data, 15);
 


That is, we use the three dimensions of the Lorenz system, where each dimension is a column and the data points are in the rows, the delay parameter is tau = 15, and the default values are used for the number of embeddings considered (1 to 10), the distance criterion Rtol (10), and plot of the function is also provided by default. Viewing the FNN-function displayed in Figure 2B, this suggests that the available three-dimensional time series is already of appropriate dimensionality, and no further time-delayed embedding is necessary.

Table 1 summarizes the results for all the possible combinations of time series of dimension 1, 2, and 3 constructed from the three variables *x*, *y*, and *z* of the Lorenz system. As can be seen, the dispersion of delays tends to decrease as the dimensionality of the data set increases, converging in average toward the delay parameter estimated for the fully three-dimensional system. Furthermore, the embedding parameter decreases as the dimensionality of the time series increases, clearly showing a dimensionality of 3 for all one-dimensional time series, and likewise a dimensionality of 3 for the three-dimensional time series. For the cases of the two-dimensional time series, we necessarily get mixed results that can at best approximate the true dimensionality of the system, because the two-dimensional time series already provides two dimensions, and there is no integer-multiple of two for a three-dimensional system. Hence, the parameter either slightly overestimates the true dimensionality (as for the combination *x* and *y*), or underestimates the true dimensionality (as for the combinations *x* and *z*, as well as *y* and *z*).

**Table 1:** Estimated delay τ and embedding dimension *D* for different combinations of time series from the Lorenz system.

**Time series**	***d***	**τ**	***D***	***D*·*d***
*x*	1	19	3	3
*y*	1	15	3	3
*z*	1	12	3	3
*x, y*	2	17	2	4
*x, z*	2	16	1	2
*y, z*	2	14	1	2
*x, y, z*	3	15	1	3

*Also shown is the dimension of the data d and the dimension of the resulting phase space D · d*.

Unlike the classical embedding parameter obtained from univariate FNN algorithms, the multidimensional FNN parameter does not provide the overall estimate of dimensionality, but has to be multiplied with the dimensionality of the underlying time series. For example for *x*, we have a one-dimensional time series and an embedding parameter of three, which provides an estimate of the true dimensionality of 1 · 3 = 3. For the combination of *x* and *y*, this estimate is 2 · 2 = 4, and for the combination of all three dimensions it is 3 · 1 = 3. To determine the necessary number of embeddings, the dimensionality now needs to be compared to the dimensionality of the data: For *x*, because it is a one-dimensional time series, the data need to be embedded two additional times. For the combination of *x* and *y*, the time series is two-dimensional and the estimate of the true dimensionality is four. Hence, the two-dimensional time series needs to be embedded one additional time. Finally, for the combination of *x*, *y* and *z*, the data are already three-dimensional to begin with, and hence do not need to be embedded any further.

The added value of using the multivariate functions over univariate for multivariate time-series becomes clear when we examine a case where we have a dimensional time series composed of dimensions that have “embeddable” dynamics and dimensions that are uninformative for embedding (i.e., random noise). Consider the case where we have a multidimensional time series with two dimensions, where one is the *x*-dimension of the Lorenz system and the other one is random noise drawn from a uniform distribution. From the perspective of an empirical scientist, this could be the case where two dimensions of a system are measured and it is (wrongly) assumed that both capture dimensions of the actual dynamics of the system, when in fact only one of these two dimensions captures the system dynamics, but the other one is completely uninformative.

If we use the univariate estimation of embedding parameters for our two-dimensional time series, we would observe the following estimates for the embedding parameters: For the first dimension of our two-dimensional time series (i.e., *x*-dimension of the Lorenz system) we would estimate τ = 19 and *D* = 3 (see also the first row of Table 1). For the second dimension of our two-dimensional time series (i.e., random numbers), we would estimate τ = 1 and *D* = 3. For empirical data, such differences are usually resolved by averaging the parameters (e.g., Wallot and Leonardi, under review), which would lead us to τ = 10 and *D* = 3.

However, because the univariate functions do not estimate the number of times one needs to embed the data *per se*, but the absolute dimensionality of the systems, we need to divide the embedding parameter by the dimensionality of our time series. That is, we conclude that we have a three-dimensional system, and assume that we have measured two out of these three dimensions. Hence, our estimate for the number of times the time series needs to be embedded is *D* = 3/2 = 1.5. Since we can embed our data only in integer dimensions, we must now choose whether we want to slightly under-embed (i.e., to not embed at all, *D* = 1) or over-embed (*D* = 2) the data. However, both estimates of underestimate the true number of times the data need to be embedded, which is 2 additional times.

Using the multivariate functions, we obtain a delay parameter of τ = 10 and an embedding parameter of *D* = 3. Here, τ is indeed underestimated as in the univariate approach, but *D* is estimated correctly, because for the multivariate estimation, *D* = 3 does not mean that the dimensionality of the system is three, but that the two-dimensionally time series needs to be embedded two additional (i.e., *D* − 1) times. Hence, the multivariate functions provide a better estimate of the embedding parameters than the univariate functions. However, the estimates are not perfect (τ is still being underestimated), and of course other scenarios are possible where the correct embedding parameters could have been obtained by chance using the univariate functions and averaging of the parameter estimates (i.e., in case the dimensionality for our random number series would have been estimated with *D* = 9).

## 5. Description of functions

The function mdDelay calculates the average mutual information for each component signal of a multivariate time series for a specified number of lags and provides the average of individual results. The function mdFnn calculates false nearest neighbors for a multivariate time series for a specified number of embeddings and provides the percentage of false nearest neighbors for each successive embedding of the multivariate time series. The inputs and outputs of the two functions are described in more detail below.

### 5.1. Inputs


mdDelay    (data)
 


*data* is an *n* × *d* matrix, where *n* is the number of data points in the time series and *d* is the number of dimensions of the time series. The function can be called using the optional parameters listed in Table 2, e.g., tau = *mdDelay* (data, “plottype,” “both”). The optional parameter “criterion” controls what method is used to find the optimal delay as follows:

**Table 2:** Optional parameters for the function mdDelay.

**Parameter**	**Description**	**Values**	**Default value**
“criterion”	The criterion used to find the delay	“firstBelow,” “localMin”	“firstBelow”
“threshold”	Value below which AMI is considered sufficiently low	real number	1/*e*
“numBins”	The number of bins used in histograms	integer	10
“maxLag”	The highest time lag used to compute AMI	integer	10
“plottype”	Controls the type of plot produced	“mean,” “all,” “both,” “none”	“mean”

“*criterion”* controls the method used for finding the optimal delay. If set to “firstBelow” the function will use the lowest delay at which the AMI function drops below the value set by the “*threshold”* parameter. If set to “localMin” the function will use the position of the first local minimum of the AMI function. If no local minimum is found the function will fall back to using “firstBelow” and if no value below “threshold” is found the function will fall back to using the position absolute minimum in the range [1, “maxLag”]. The default value is: “firstBelow.”


mdFnn(data,  tau)
 


*data* is an *n* × *d* matrix, where *n* is the number of data points in the time series and *d* is the number of dimensions of the time series.

*tau* is an integer that gives the delay (number of lags) for the embedding. There is no default value for this input.

The function can be called using the optional parameters listed in Table 3, e.g., *mdFnn*(data, tau, “maxEmb,” 20, “doPlot,” false) to set the maximum embedding dimension to 20 instead of the default value of 10, and turn off plotting of the result. The parameters that can be set are:

**Table 3:** Optional parameters for the function mdFnn.

**Parameter**	**Description**	**Values**	**Default value**
“maxEmb”	The maximum number of embedding dimensions	Integer	10
“doPlot”	Controls whether results are plotted	Integer/logical	1/true
“numSamples”	Number of randomly sampled points	Integer	500
“Rtol”	First criterion for FNN classification	Real number	10
“Atol”	Second criterion for FNN classification	Real number	2

“*numSamples”* is the number of randomly selected coordinates from phase-space for which (false-)nearest neighbors are computed. Selecting a random sample of coordinates is done to decrease computation time for long time series. The default value is 500, or alternatively the maximum number of available phase-space coordinates if that number is less than 500.

“*Rtol”* provides the first criterion for classifying neighboring coordinates in phase-space as false neighbors. The default value is 10.

“*Atol”* provides the second criterion for classifying neighboring coordinates in phase-space as false neighbors. The default value is 2.

### 5.2. Outputs


tau = mdDelay(data)
 


The returned value *tau* is the estimate of the time delay to be used for the embedding of *data*. The user is stronlgy urged not to use this estimate without checking the plotted AMI that is also provided by the function. It is also advisable to check if this value depends on the method and parameters (e.g., *maxLag, numBins, threshhold*) used.

 
[fnnPerc, embTimes] = mdFnn(fnnPerc, embTimes)
 


*fnnPerc* is a vector with the percentage of points classified as false nearest neighbors for each (additional) embedding of the time series.

*embTimes* is a vector providing the number of times the *d*-dimensional signal has been embedded.

## 6. Discussion

In this paper, we provide two methods—and their implementations in Matlab—for the estimation of embedding parameters of multivariate time series. These methods make it possible to estimate embedding parameters for analysis techniques that require phase-space reconstruction when multidimensional time series have been recorded (e.g., Wallot et al., 2016b; Wallot, 2018), the analysis of which has become increasingly prominent in physiological research that considers the integration of multiple dependent measures to capture arousal, for example, or in joint action research where groups of participants interact and continuous measures of each participant are recorded.

We want to point out some limitations of the methods presented here. First of all our algorithms to compute estimates for the time delay and embedding dimension should not be used without inspecting the plots, since both the choice of time delay and embedding dimension rely on the form of the curves (AMI and FNN). As an example, the estimated time delay for the *x* variable of the Lorenz system in our example is τ = 19, which is based on the first local minimum of the AMI function. However, inspection of the mutual information as a function of time delay reveals that the curve is very flat in the range τ ∈ [13, 20], so it is probably better to select a value from the lower end of this interval, since we are interested in the lowest value of τ that minimizes the mutual information. Given a sufficient number of data points, the lowest value for the mutual information function could be chosen, of course, but higher values for τ (and *D*) decrease the number of data points available for analysis, and hence it might be important to select lower values for these parameters within a reasonable range.

Another limitation is that we only consider a fixed bin width when we construct the histograms used to compute mutual information. For time series that have very different densities in different parts of the range, adaptive binning algorithms may be more appropriate (see Cellucci et al., 2005, for a comparison of algorithms).

If the individual dimensions have very different AMI functions and result in very different estimates of the time delay, it may be inappropriate to embed them all with the same value of τ. In some cases this may be handled by re-sampling some of the dimensions at a lower rate, but care must be taken not to lose relevant information in the process. A better approach might be to use different time delays for the individual dimensions when performing the phase space embedding.

A practical issue for long, high-dimensional time series is the computation time for the estimation procedure which might be several minutes per time series. Here, drawing random sub-series from the original time series and estimating dimensionality over several such sub-series can provide a solution to this problem, or at least a substantial decrease in processing time.

Even though our methods allow for the estimation of embedding parameters of multidimensional time series, they provide limited accuracy when the true dimensionality of the system under investigation is not an integer-multiple of the dimensionality of the time series, because the method of time-delayed embedding proceeds only in integer dimensions. However, in the examples shown above, our method results in the minimum possible error for correct data sets, since the resulting phase-space dimension differs from the correct dimension by one, and in improved estimates for noisy/ill-composed data sets, where estimates—particularly of dimensionality—are better compared to univariate parameter estimation.

## Author contributions

SW contributed the concept of the study. SW and DM designed the study, implemented the software solution and wrote the manuscript.

### Conflict of interest statement

The authors declare that the research was conducted in the absence of any commercial or financial relationships that could be construed as a potential conflict of interest.

## Supplementary material

The Matlab functions described here are available on GitHub (github.com/danm0nster/mdembedding) along with examples and the code used to produce the figures in this article.

## Appendix I: Abbreviations and notation

· – The dot (as in $\overset{\cdot }{x}$) denotes the time derivative $\overset{\cdot }{x}=\frac{\text{d}x}{\text{d}t}$

AMI – Average mutual information or Auto Mutual Information, see *I*(*x*(*t*), *x*(*t* + τ))

CRQA – Cross-Recurrence Quantification Analysis

*d* – dimensionality of a multivariate time series

*D* – Embedding parameter for phase-space reconstruction

FNN – False-nearest neighbor(s)

*I*(*x*(*t*), *x*(*t* + τ)) – Mutual information. A nonlinear measure of dependence between (*x*(*t*) and *x*(*t* + τ))

MdRQA – Multidimensional Recurrence Quantification Analysis

*p*_*i*_ – The probability that a point sampled from *x*(*t*) is in bin number *i* of the histogram of *x*

*p*_*ij*_(τ) – The probability that *x*(*t*) is in bin *i* and *x*(*t* + τ) is in bin *j*

*R* – distance between a coordinate and its nearest neighbor in phase-space

*R*_tol_ – distance criterion for defining false nearest neighbors

RQA – Recurrence Quantification Analysis

*x* – A 1-dimensional time series. Also: *x*-dimension of the Lorenz system

*y* – *y*-dimension of the Lorenz system

**y** – Phase-space embedded time series constructed from lower dimensional data

β – Coupling parameter (beta) for the Lorenz system

ρ – Coupling parameter (rho) for the Lorenz system

σ – Coupling parameter (sigma) for the Lorenz system

τ – Delay parameter (tau) for phase-space reconstruction

*z* – z-dimension of the Lorenz system
